# Functional and Patient-Reported Outcomes of RegJoint™ Interpositional Arthroplasty for Thumb Carpometacarpal Joint (CMCJ) Arthritis: A Two-to-Five-Year Follow-Up Study

**DOI:** 10.7759/cureus.88064

**Published:** 2025-07-16

**Authors:** Raheel Faiz, Raja Babar Akram, FR Hashmi

**Affiliations:** 1 Orthopaedics and Traumatology, South Warwickshire University NHS Foundation Trust, Warwickshire, GBR

**Keywords:** arthroplasty, carpometacarpal arthritis, interpositional, regjoint™, thumb

## Abstract

Background/purpose

This study aimed to assess the long-term outcomes of patients treated with RegJoint™ interpositional arthroplasty for thumb carpometacarpal joint (CMCJ) arthritis as a replacement to traditional practices after a follow-up period of two to five years (mean of 42 months).

Methods

A retrospective cohort study was conducted, which included 35 consecutive patients who underwent the RegJoint™ interpositional arthroplasty between 2017 and 2022 at the Department of Orthopaedics, South Warwickshire University NHS Foundation Trust, Warwickshire, Great Britain. Twenty-five patients were included in this study who met the inclusion and exclusion criteria and who agreed to participate in the study. Functional outcomes, including range of motion, quality of life, and patient satisfaction, were evaluated using standardised assessments such as the QuickDASH score and the Thumb Disability Examination (TDX) score.

Results

Among the 25 patients included in the study, the mean age was 62 years (range: 48-79 years), with a mean follow-up duration of 42 months (range: 24-60 months). The study demonstrated favourable postoperative outcomes, with a mean QuickDASH score of 15.8; some patients achieved complete symptom resolution. Notably, there were no reported cases of abnormal reactions, infections, or revisions within this cohort.

Conclusion

This long-term follow-up study demonstrates that RegJoint™ interpositional arthroplasty can be considered for patients with thumb CMCJ arthritis, given the good patient satisfaction and low complication rates. This technique provides a mean of alternative to traditional surgical techniques, particularly in patients wanting joint-preserving solutions.

## Introduction

RegJoint™ interpositional arthroplasty represents a joint-preserving alternative in the surgical treatment of thumb carpometacarpal joint (CMCJ) osteoarthritis [1]. Traditional procedures such as trapeziectomy and ligament reconstruction techniques effectively relieve pain but may lead to reduced thumb length, grip strength, or prolonged recovery [2,3]. Total joint replacements preserve motion but carry risks of implant failure, while arthrodesis eliminates pain at the cost of mobility [4].

Synthetic interposition implants attracted interest among hand surgeons over the last decade, but none achieved consistently satisfactory outcomes [4]. Complications such as reactive synovitis, osteolysis, foreign-body reaction, and spacer subluxation remain common reasons why synthetic devices have not become mainstream [4].

RegJoint™ spacer is a porous biodegradable poly-L/D-lactic acid copolymer (PLDLA, 96 % L / 4 % D) [1]. It functions as a temporary porous spacer with rapid absorption, facilitating scar tissue ingrowth and development of a dense fibrous pseudoarthrosis in the articular space with no permanent foreign material remaining. Resorption of PLDLA is completed in approximately two to three years [5]. Histologically, fibroblasts infiltrate the spacer, followed by capsular formation and development of fibrous tissue extending to the centre of the implant.

RegJoint™ offers a minimally invasive option that maintains joint space and kinematics with limited bone resection [6]. Its biodegradable, porous structure supports fibrous tissue ingrowth and provides cushioning without complications associated with metal or non-degradable polymer implants [6,7]. Importantly, it does not preclude later conversion to standard procedures if required [7]. Positioned between trapeziectomy and total joint replacement, RegJoint™ provides a biologically integrated, motion-preserving solution.

This study aims to assess the real-world effectiveness of RegJoint™ by analysing patient‑reported outcomes over a two-to-five-year follow-up period (mean of 42 months). Functional recovery was evaluated using the QuickDASH [8,9] and the Thumb Disability Examination (TDX) [10,11] scores, offering insights into postoperative disability, pain, and patient satisfaction. Complications, including infection, adverse reactions, and symptomatic osteolysis, were also examined.

## Materials and methods

Study design

A retrospective cohort study was conducted, which included 35 consecutive patients who underwent RegJoint™ interpositional arthroplasty for thumb CMCJ osteoarthritis between 2017 and 2022 under a single surgeon locally at the Department of Orthopaedics, South Warwickshire University NHS Foundation Trust, Warwickshire, Great Britain. 

Patient selection and preoperative analysis

Inclusion Criteria

The inclusion criteria included patients with isolated CMCJ arthritis, a minimum follow-up of two years, and willingness to complete patient-reported outcome measures (PROMs).

Exclusion Criteria

The exclusion criteria are a follow-up of less than two years, inflammatory arthritis, significant adjacent joint disease requiring intervention, or prior CMCJ surgery.

After exclusions, 25 patients met the criteria and were assessed via the clinic.

Out of 10 of those who were excluded, four had early scaphoid (STT) arthritis, five had previous CMCJ surgery, and one was lost to follow-up.

Surgical technique and postoperative protocol

**Figure 1:**
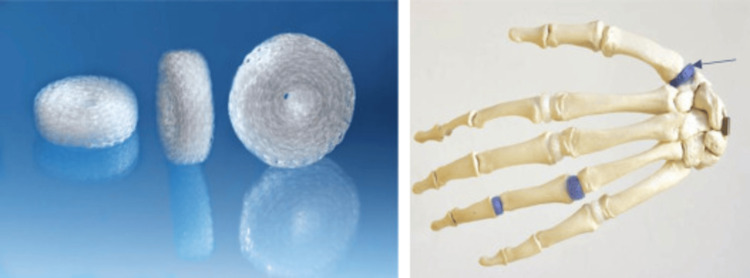
RegJoint™ spacer

All procedures were performed using a dorsal approach, standard interpositional arthroplasty technique, partially excising the trapezium and inserting the RegJoint™ scaffold [1] (Figure 1) within the joint space.

General anaesthesia was used for all patients. A tourniquet was also used. All were day cases. 

We implemented an antibiotic prophylaxis protocol where one dose of intravenous (IV) antibiotics (Gentamicin and Flucloxacillin) was administered prior to the beginning of surgery. Teicoplanin was used in patients who were penicillin allergic.

Postoperative rehabilitation involved a staged approach to protect the joint, support tissue integration, and restore function. Initial immobilisation in a thumb spica back slab was maintained for the first two weeks, followed by gradual introduction of thumb range of motion exercises from week 2 to week 6. Strengthening and functional tasks began after six weeks, with most patients returning to normal activities by 12 weeks.

Outcome measures

Patient-reported outcomes were assessed using the following: 1) The QuickDASH score is a validated patient-reported outcome measure assessing upper limb function and symptoms. It consists of 11 core items [8,9] and optional modules for work, sports, or music, with all items rated on a five-point scale. Scores range from 0 to 100, which measures upper limb disability (scale 0-100, higher score = worse function). 2) The Thumb Disability Examination (TDX) is a validated patient-reported outcome measure consisting of 20 items assessing pain, impairment, activity limitation, and satisfaction related to thumb function [10,11]. It can be scored in total or by subsection, with higher scores indicating greater disability and a maximum score of 100. A validated thumb-specific PROM assessing pain, function, and satisfaction (scale 0-100, higher score = worse function).

Complications and revision rates were recorded.

Statistical analysis

Descriptive statistics were used to summarise patient demographics and postoperative outcomes. Means and ranges were calculated for continuous variables such as QuickDASH and TDX scores, while categorical variables such as sex distribution were reported as percentages. All analyses were performed using Microsoft Excel (Microsoft Corporation, Redmond, WA, USA).

## Results

Patient demographics

A total of 25 patients were included in the study, comprising eight males (32%) and 17 females (68%). The mean age at the time of surgery was 62 years (range: 48-79 years). The mean follow-up duration was 42 months (range: 24-60 months).

Imaging

**Figure 2:**
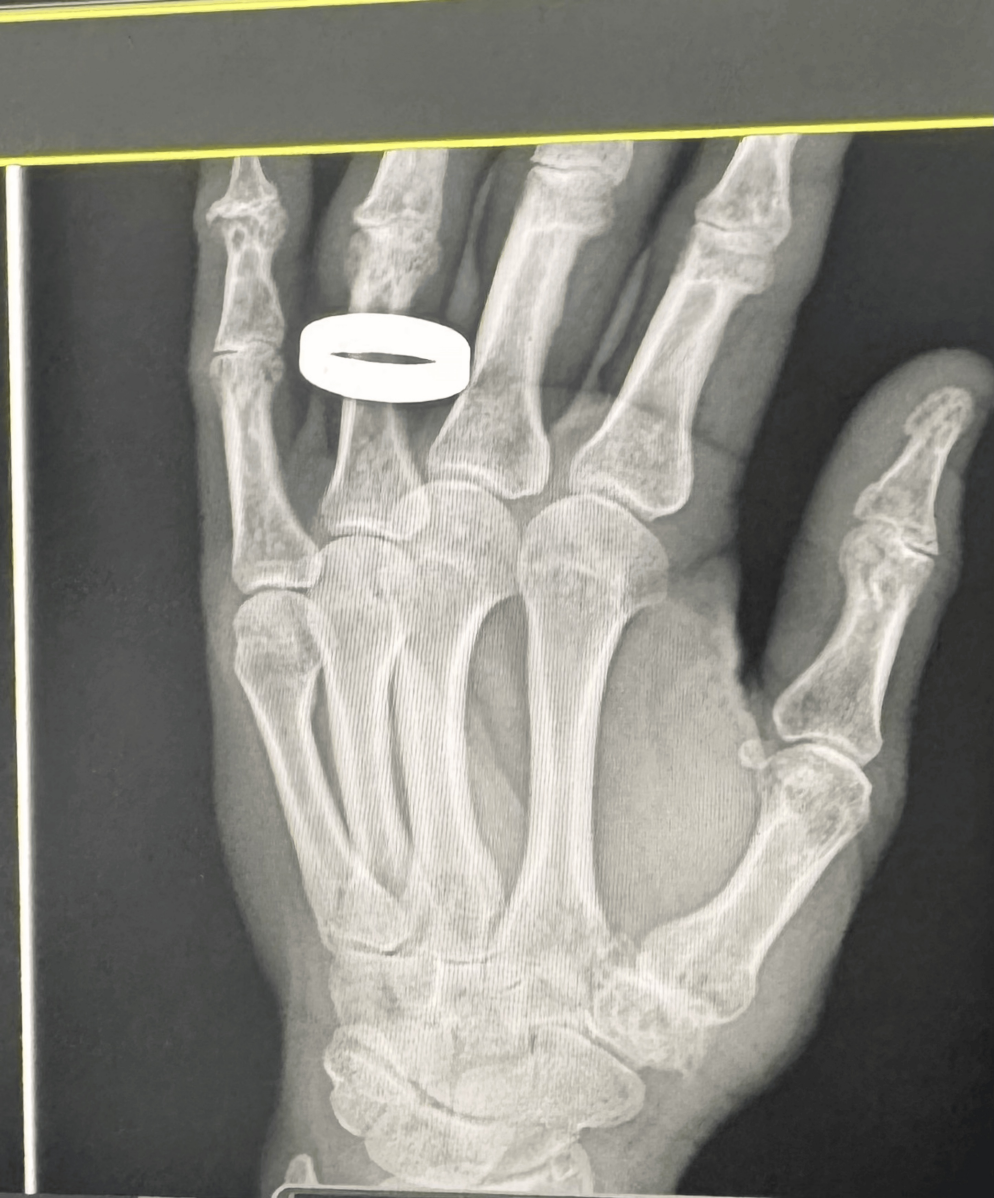
Preoperative thumb carpometacarpal joint (CMCJ) showing loss of joint space

**Figure 3:**
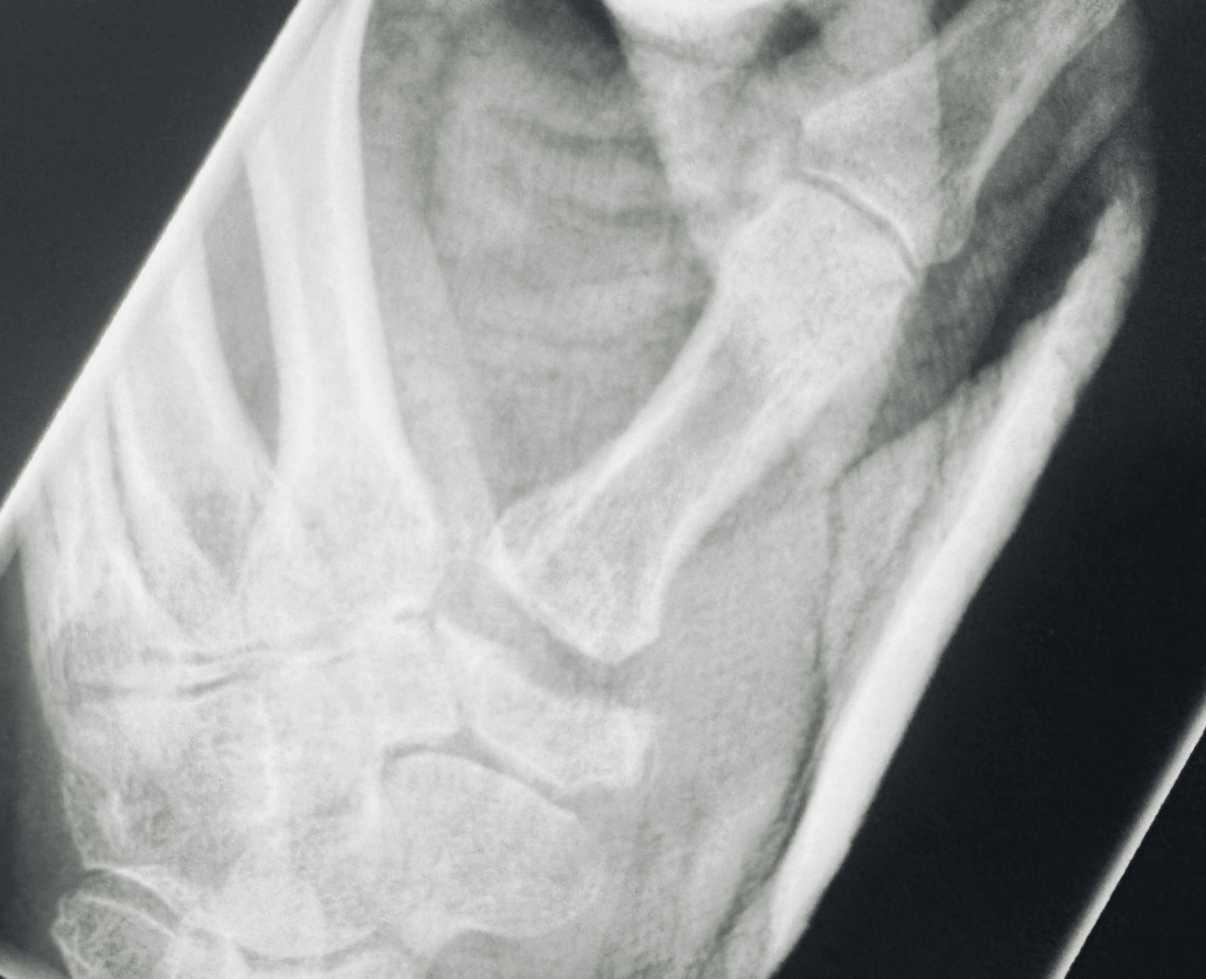
Postoperative two-week follow-up of thumb carpometacarpal joint (CMCJ) with increasing joint space

**Figure 4:**
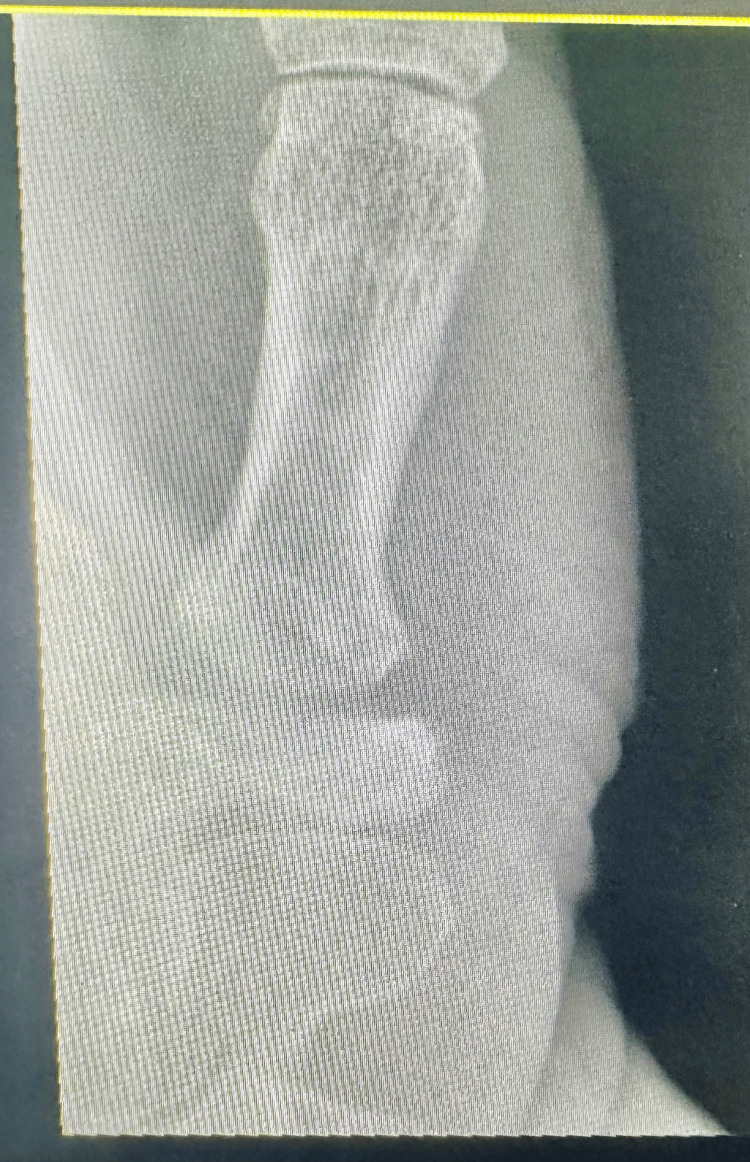
Postoperative two-year follow-up of thumb carpometacarpal joint (CMCJ)

Figure 2 shows the preoperative image. Figures 3-4 show the postoperative follow-up images at two weeks and two years, respectively.

Functional outcomes

Functional outcome scores are summarised in Table 1. The mean QuickDASH score was 15.8 (range: 0-43), indicating mild functional impairment postoperatively (Table 1). The mean TDX score was 16.83 (range: 0-43.75), reflecting low residual disability (Table 1).

**Table 1:** Functional outcomes

Outcome measure	Mean score	Range
QuickDASH	15.8	0 - 43
TDX	16.83	0 – 43.75

Postoperative complications and revisions

No wound complications, infections, or foreign body reactions were reported during the follow-up period. There were no cases requiring revision surgery or conversion to trapeziectomy, supporting the safety and durability of the procedure.

## Discussion

Several studies have investigated the outcomes of RegJoint™ interpositional arthroplasty for thumb CMCJ osteoarthritis, highlighting generally positive clinical results with some variation in technique and follow-up duration [12]. Kennedy et al. reported satisfactory functional outcomes and no significant adverse effects over a minimum two-year follow-up, suggesting the implant’s safety and efficacy in preserving thumb function [13]. Sander et al. combined RegJoint™ with a modified APL suspension technique and observed pain relief, improved strength, and preserved mobility, supporting its role as a simplified and effective procedure. Similarly, Marcuzzi et al. [14] found long-lasting pain relief and functional gains, despite radiographic evidence of metacarpal collapse, affirming RegJoint™ as a viable alternative to traditional trapeziectomy with tendon interposition [15]. Vasileva et al. noted substantial improvements in pain, swelling, and function within one year, emphasising the importance of postoperative physiotherapy. However, a systematic review by Spiteri and Giele [16] raised concerns about higher complication rates associated with poly-l/d-lactide scaffolds like RegJoint™, calling for more extensive, long-term studies to better define its durability and long-term benefits. Collectively, the evidence supports the potential of RegJoint™ as a joint-preserving option, although further comparative and longitudinal research is warranted. 

The findings of this study support RegJoint™ interpositional arthroplasty as a viable option for patients with thumb CMCJ arthritis, offering good functional outcomes and a low complication rate over a mid-term follow-up. Compared to earlier studies by Mattila et al. [4], which reported concerns regarding foreign body reactions and osteolysis, our cohort experienced no such complications, likely due to refined surgical techniques, careful patient selection, and improved post-operative rehabilitation. These results align with those of Kennedy et al. [12], further reinforcing RegJoint™ as a safe and effective alternative to total trapeziectomy. In addition, a pilot study conducted in Poland by Florek et al. [17] demonstrated that the use of RegJoint™ implants had less pain, better limb performance, better quality of life, and higher values for hand-grip strength. Key advantages of RegJoint™ include joint preservation by avoiding complete trapeziectomy, the benefits of a biodegradable scaffold promoting soft tissue ingrowth, a low revision rate with no cases requiring conversion to trapeziectomy or total joint replacement, and faster recovery times compared to ligament reconstruction techniques.

However, the limitations of this study include the absence of radiographic follow-up, highlighting the need for future MRI/X-ray assessments to evaluate bone adaptation. Because this was a retrospective study, we did not have access to systematically collected preoperative patient-reported outcomes (PROMs), which limits our ability to assess the subjective impact of complications on PROMs. A relatively small sample size necessitates larger multi-centre studies, and the lack of a comparative control group suggests that further trials should directly compare RegJoint™ with alternative procedures such as pyrocarbon implants or ligament reconstruction techniques.

## Conclusions

Overall, RegJoint™ interpositional arthroplasty has demonstrated positive functional outcomes, good patient satisfaction, and low complication rates, especially in CMCJ application. While early concerns about foreign body reactions and osteolysis were raised, recent studies suggest these are less of an issue with refined surgical techniques. The implant remains a viable alternative to metal-based implants and traditional surgical techniques, particularly in patients seeking joint-preserving solutions; thus, its use should be considered on a case-by-case basis.
